# Evaluation of fluence‐based dose delivery incorporating the spatial variation of dosimetric leaf gap (DLG)

**DOI:** 10.1120/jacmp.v17i1.5883

**Published:** 2016-01-08

**Authors:** Lalith K. Kumaraswamy, Zhengzheng Xu, Daniel W. Bailey, Jonathan D. Schmitt, Matthew B. Podgorsak

**Affiliations:** ^1^ Department of Radiation Medicine Roswell Park Cancer Institute Buffalo NY; ^2^ Department of Cell Stress Biology Roswell Park Cancer Institute Buffalo NY; ^3^ Department of Physiology and Biophysics State University of New York at Buffalo Buffalo NY; ^4^ Department of Radiation Oncology Northside Hospital Atlanta GA; ^5^ Department of Radiation Medicine RadAmerica, LLC‐MedStar Health Baltimore MD USA

**Keywords:** dosimetric leaf gap, dose calculation, treatment planning systems, multileaf collimators

## Abstract

The Eclipse treatment planning system uses a single dosimetric leaf gap (DLG) value to retract all multileaf collimator leaf positions during dose calculation to model the rounded leaf ends. This study evaluates the dosimetric impact of the 2D variation of DLG on clinical treatment plans based on their degree of fluence modulation. In‐house software was developed to retrospectively apply the 2D variation of DLG to 61 clinically treated VMAT plans, as well as to several test plans. The level of modulation of the VMAT cases were determined by calculating their modulation complexity score (MCS). Dose measurements were done using the MapCHECK device at a depth of 5.0 cm for plans with and without the 2D DLG correction. Measurements were compared against predicted dose planes from the TPS using absolute 3%/3 mm and 2%/2 mm gamma criteria for test plans and for VMAT cases, respectively. The gamma pass rate for the 2 mm, 4 mm, and 6 mm sweep test plans increased by 23.2%, 28.7%, and 26.0%, respectively, when the measurements were corrected with 2D variation of DLG. The clinical anal VMAT cases, which had very high MLC modulation, showed the most improvement. The majority of the improvement occurred for doses created by the 1.0 cm width leaves for both the test plans and the VMAT cases. The gamma pass rates for the highly modulated head and neck (H&N) cases, moderately modulated prostate and esophageal cases, and minimally modulated brain cases improved only slightly when corrected with 2D variation of DLG. This is because these cases did not employ the 1.0 cm width leaves for dose calculation and delivery. These data suggest that, at the very least, the TPS plans with highly modulated fluences created by the 1.0 cm fields require 2D DLG correction. Incorporating the 2D variation of DLG for the highly modulated clinical treatment plans improves their planar dose gamma pass rates, especially for fields employing the outer 1.0 cm width MLC leaves. This is because there are differences in DLG between the true DLG exhibited by the 1.0 cm width outer leaves and the constant DLG value modeled by the TPS for dose calculation.

PACS numbers: 87.55.D, 87.55.Qr, 87.56.Fc, 87.56.N, 87.56.nk

## I. INTRODUCTION

The use of multileaf collimators (MLCs) is instrumental in enabling radiation therapy to deliver conformal dose via intensity modulated radiation therapy (IMRT) and volumetric modulated arc therapy (VMAT).^1^, ^2^, ^3^, ^4^, ^5^ These techniques deliver dose distributions by the complex motion of the MLC. The magnitude of the complexity of the dynamic MLC motion depends on the location of the target and the organs at risk. The Eclipse treatment planning system (TPS) (Varian Medical Systems, Palo Alto, CA) must accurately model these complex motions of the MLC to deliver the intended dose according to the prescribed dose constraints. It is crucial to model accurately the MLC characteristics defined in the TPS, such as the dosimetric leaf gap (DLG), in order to deliver the intended dose to patients.

Varian linear accelerators have rounded MLC leaf ends to achieve better off‐axis dosimetric properties. Consequently, some radiation passes between the leaves, even through completely closed leaf pairs. The TPS approximates the MLC as straight edged and takes into account the actual rounded leaf tip transmission by pulling the leaf tip back by half the value of the DLG during optimization and dose calculation so the modeled gap between the fully closed leaf pair equals the DLG.^6^, ^7^

**Figure 1:**
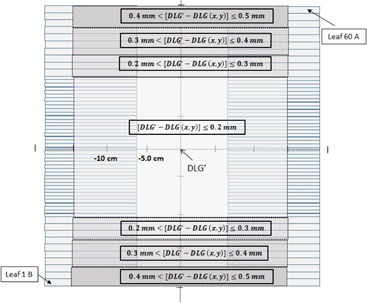
Illustration showing the difference in DLG with respect to DLG measured at CAX (DLG′) for all leaf pairs. The DLG′ is measured for the middle pair MLCs (Leaf #30) at CAX. The DLG (x,y) is measured for each leaf pair from 13.0 cm off‐axis distance to central line.

Currently, the TPS models the dynamic MLC motion for all the MLC leaf pairs, utilizing only the DLG measured under the middle pair of MLC leaves at central axis (CAX). However, it has been shown that there is a variation of DLG among all individual Millennium 120 (Varian Medical Systems, Palo Alto, CA) MLC leaf pairs.^(7)^ The Millennium 120 MLC consists of 60 pairs of leaves. The central 40 leaf pairs (spanning from CAX to 10 cm off axis) have a projected isocenter width of 0.5 cm, whereas leaf pairs residing at greater than 10 cm off‐axis have a 1.0 cm width projected at isocenter. It was found that the variation in DLG is most pronounced between the 0.5 cm width group of leaves and the 1.0 cm width group of leaves (mean and maximum variation of 0.32 mm and 0.65 mm, respectively).^(7)^ Based on these findings, Fig. 1 groups the MLC travel span based on the differences between the measured DLG for the leaf pair located in that region and the DLG measured at CAX (DLG'). The DLG among the middle 0.5 cm width leaves do not vary much in their respective travel span. The outer 1.0 cm width leaves, however, have much lower DLG values compared to the middle MLC leaf pair, about 0.3 mm less and 0.6 mm less for the inner most 1.0 cm width leaves and for the outer most 1.0 cm width leaves, respectively.

Several investigators have shown that intraleaf MLC transmission is a crucial factor in determining DLG.^7^, ^8^ Intraleaf transmission is a varying parameter at off‐axis distances from CAX,^9^, ^10^ causing the DLG to also vary proportionally at off axis distances.^(7)^ The outer 1.0 cm width leaf pairs have lower DLG values because the intraleaf transmission through the central 0.5 cm width leaves is greater than the outer 1.0 cm width leaves.^(7)^

This 2D variation in DLG could impact the delivered dose at off‐axis distances. Oliver et al.^(11)^ systematically looked at the MLC positional errors for several clinical VMAT cases. They investigated three types of MLC errors: random (Type 1), systematic errors in MLC position in the same direction (Type 2), and systematic errors in MLC position in the opposite direction (Type 3). They found that Type 3 errors, which can represent DLG errors since the MLCs are retracted in the opposite direction, produced the largest change in the cumulative dose distribution. For PTV70 (H&N), the dose sensitivity to Type 3 errors for D95% was 3.2%/mm for RapidArc, 2.9%/mm for simple IMRT plans, and 9.6%/mm for complex IMRT plans. LoSasso et al.^(8)^ showed that a difference of 0.5 mm between the DLG in the TPS and the true DLG exhibited by the MLC leaf pair can give rise to a dose error of at least 2.0% for DLMC fields less than 2.5 cm aperture width. Rangel and Dunscombe^(12)^ showed that a systematic MLC gap change of 0.6 mm introduces an approximate 2% change in the equivalent uniform dose of the clinical target volume for a typical H&N IMRT plan. Lee et al.^(13)^ demonstrated that a maximum dose difference of up to 30.8% can be seen between TPS calculated and measured doses for inner organs at risk when the measured DLG value differs by 1.0 mm from the DLG value in TPS.

With increased popularity of image guided radiotherapy (IGRT) and VMAT, treatment fields are becoming increasingly complex, with higher modulation to deliver highly conformal dose to the target while sparing organs at risk. Highly modulated treatment fields require complex MLC motion. Therefore, small variations in MLC gaps (DLGs) could result in large differences between the TPS calculated dose and the dose delivered to the patient. Webb^(14)^ and Nauta et al.^(15)^ introduced metrics to characterize the degree of modulation of an IMRT field based on fluence modulation. Nauta and colleagues^(15)^ found that pretreatment quality assurance (QA) pass rates as measured by the MapCHECK (Sun Nuclear Corp, Melbourne, FL) were lower for treatment fields with high fluence modulation, as compared to treatment fields with moderate fluence modulation. McNiven et al.^(16)^ developed a simple, but effective, metric called Modulation Complexity Score (MCS), based on aperture area variability of successive control points and leaf sequence variability among individual MLC leaves, to assess the complexity of step‐and‐shoot IMRT fields. Masi et al.^(17)^ further developed this score to evaluate the complexity of VMAT fields. They found that pretreatment IMRT/VMAT QA pass rates were lower for fields with low MCS (high modulation) and higher for fields with high MCS (low modulation).

Our study aims to incorporate the 2D variation of DLG into several clinical treatment plans using in‐house developed software. The 2D DLG‐corrected and uncorrected plans are compared to respective measurements to validate the importance of incorporating the 2D variation of DLG in the TPS. The degree of improvement in pass rate for each 2D DLG‐corrected treatment plan is assessed as a function of the level of modulation of the treatment fields represented by the MCS.

## II. MATERIALS AND METHODS

### A. LINAC and MLC

The DLG values for 6 MV photon beam were measured on two Varian Trilogy linear accelerators (Varian Medical Systems, Palo Alto, CA) equipped with Millennium 120 MLC, as described previously.^(7)^

### B. 2D variation of DLG

The DLG values for all 60 MLC leaf pairs were measured with an increment of 1.0 cm in the leaf travel direction. Measurements were performed with a MapCHECK diode array system. Multiple sliding window fields of different gap widths were used to derive the 2D map of DLGs, as described in our previous work.^(7)^

After determining the DLGs at each off axis points for all the MLC leaf pairs, a matrix of correction factors containing the differences in DLG from CAX at each cell was created. The dimension of the matrix is 60 rows (60 MLC leaf pairs) by 27 columns (off‐axis distance from ‐13cm to +13cm with increment of 1.0 cm).

### C. 2D DLG correction

**Figure 2:**
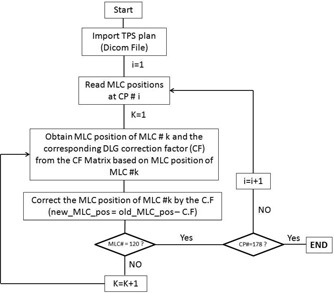
The flowchart of the in‐house software developed to modify the TPS plan with 2D variation of DLGs.

The TPS applies a constant DLG value for all the MLC leaves during dose calculation. Since there is a variation in DLG among leaf pairs, the delivered dose distribution will be from the MLC leaf positions that exhibit varying DLG among MLC leaf pairs. Hence the calculated dose distribution (constant DLG) from the TPS may not be the same as the delivered dose distribution (varying DLG). The MLC leaf positions from the TPS plan should be modified in such a way that will result in a delivered dose distribution from a constant DLG value for all leaf pairs. In order to incorporate the 2D variation of DLG into delivery, an in‐house software application was developed. This software imports the TPS plan with all the control point sequences. This software does not modify the planned dose in the TPS. It alters the MLC positions at each control point of the delivered plan, based on the 2D variation map of the DLG, so the delivered plan will have MLC leaf positions that will result in a constant DLG delivery. Therefore, the resulting measured dose distribution will be from a plan based on constant DLG value, precisely like the predicted dose distribution from the TPS. For example, if the DLG value in the TPS is 0.16 cm and the measured DLG for leaf 58 at 10.0 cm off axis is 0.12 cm, the software will retract leaf 58 so the gap between leaf 58 A and 58 B increases by 0.04 cm at the 10.0 cm off‐axis distance to give an effective DLG of 0.16 cm at that location. Essentially, the MLC leaf positions from the imported TPS plan will be modified for all leaves at all locations to give an effective constant delivered DLG for all MLC leaves at all locations that matches the treatment planning system. The software exports the modified plan as a DICOM file to be delivered at the treatment console. The flowchart of the software is shown in Fig. 2.

### D. Constant gap dynamic MLC test plans

To test and validate the 2D DLG correction, several sliding window MLC gap files with gap ranging from 2 mm to 10 mm, were created and calculated in the TPS. The MLC leaf pairs for these gap fields start at CAX and slide with constant gap width, speed, and dose rate to an off axis distance of 15.0 cm. The gap widths represent various levels of modulation. The 2 mm gap plan represents high modulation since a typical highly modulated plan consists of very small MLC gaps, whereas the 10 mm gap plan represents a minimally modulated TPS plan.

### E. Clinical VMAT plans

A total of 61 clinically treated VMAT treatment fields, varying in the level of modulation, were randomly chosen for this study. The sample included large anal treatment fields with very high modulation employing almost all the 60 MLC leaf pairs down to small brain fields with very low modulation employing only the middle 0.5 cm width leaves (40 MLC leaf pairs). Table 1 shows the detailed description of the type of clinical treatment fields used in this study. All the treatment fields employed all the 40 middle 0.5 cm width leaf pairs. The anal cases used majority of the 1.0 cm width MLC leaf pairs with very high modulation to deliver conformal dose, while the H&N fields employed the innermost 1.0 cm width MLC leaf pairs with very high modulation to deliver conformal dose. The prostate, esophagus, and brain fields did not utilize the 1.0 cm width leaves to deliver conformal dose to each target. The prostate and the esophagus fields were moderately modulated, whereas the brain fields had dynamic MLC with minimal modulation. The degree of modulation is determined by calculating the MCS value for each VMAT field. A detailed description of the MCS score and its calculation methodology can be found elsewhere.^16^, ^17^

The TPS uses a constant DLG value which, in our case, has a value of 0.147 cm, and was applied to the dose calculation of all the dynamic plans. The plans were exported from the TPS and a copy of each plan was run through the in‐house software to modify the MLC positions based on the measured 2D DLG correction map. MapCHECK was employed to obtain the measurements for both sets of plans (2D‐corrected and standard plans). All measurements were obtained at 5.0 cm depth. The measured dose resulting from 2D‐corrected and standard plans was compared against the TPS predicted dose.

**Table 1:** Clinical treatment fields used to evaluate the effectiveness of the 2D DLG correction. Treatment fields were selected to include variation in the level of modulation indicated by the MCS value and number of 1.0 cm width leaves involved in creating the fluence.

*Treatment Site*	*Number of Fields*	*Level of Modulation (MCS Value)*	*Average Number of 1.0 cm Width Leaf Pairs*
Anal	19	High (0.124)	15
H&N	13	High (0.158)	4
Prostate	10	Moderate (0.219)	0
Esophagus	10	Moderate (0.245)	0
Brain	9	Low (0.350)	0

## III. RESULTS

### A. Constant gap dynamic MLC Plans (D in Material & Methods section)

**Figure 3:**
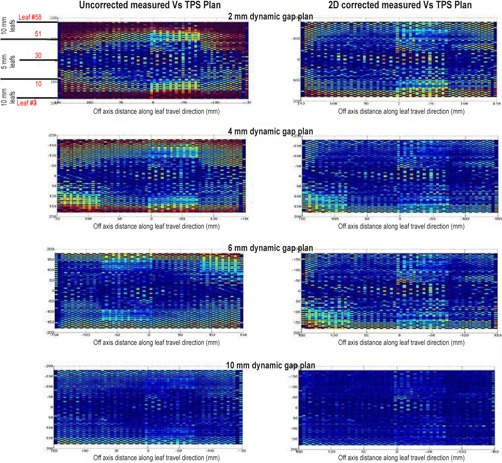
2D gamma index map comparison between the 2D DLG‐corrected measurement versus TPS plan (right) and uncorrected measurement versus TPS plan (left) for dynamic gap plans ranging from 2.0 mm to 10.0 mm gap.

The 2D DLG‐corrected plan file and the standard uncorrected plan file for the constant gap dynamic plans were measured with the MapCHECK device. The resulting dose distributions from both sets were compared with the respective predicted dose distribution from the TPS. Table 2 shows the resulting absolute gamma pass rates using 3%/3 mm criteria and a 10% dose threshold. For small dynamic gap fields (2 mm, 4 mm, and 6 mm), the pass rate increases significantly when corrected with 2D variation of DLG. For the largest gap size studied, the improvement in pass rate is less (7.7%), but still significant. Figure 3 shows the 2D map of the gamma index, where the blue dots represents detectors passing the gamma criteria of 3%/3 mm and red dots represent the detectors failing the gamma criteria for both 2D DLG‐corrected and uncorrected measurements compared to TPS prediction. This plot shows where the detectors are passing and failing in relation to MLC number and position. From Table 2 and Fig. 3, the number of detectors passing the gamma criteria for both corrected and uncorrected measurements for the 2 mm dynamic plan is very low. The higher dynamic gap fields (4 mm and 6 mm) shows a good pass rate for the corrected field (>80%), whereas the uncorrected fields show low pass rates. The pass rates for the uncorrected and corrected 10 mm dynamic gap fields are good (>90%), indicating that the 2D DLG correction becomes less significant for large dynamic gap fields. Figure 3 shows that most of the failures occur where the 1.0 cm MLC width leaves reside. This is because the 1.0 cm leaves are characterized by much lower DLG values than the 0.5 cm leaves. The 2D DLG‐corrected measurements were conducted with MLC leaf positions altered to give a constant DLG value that is represented in the TPS. As a result, the 2D DLG‐corrected measurements agree very well with the TPS‐predicted dose, even for 1.0 cm MLC leaves.

**Table 2:** Gamma pass rate (3%/3 mm) comparison between the 2D DLG‐corrected and uncorrected measured dose vs. the TPS‐predicted dose.

*Dynamic Gap Size*	*Uncorrected (%)*	*2D DLG Corrected (%)*	*Improvement (%)*
2 mm	39.1	62.2	23.2
4 mm	52.1	80.7	28.7
6 mm	66.4	92.4	26.0
10 mm	90.2	97.9	7.7

### B. Clinical VMAT plans (E in Materials & Methods section)

The measured dose distributions of the 2D‐corrected and uncorrected VMAT plans with MapCHECK were compared with the predicted dose distribution from the TPS. A 2%/2 mm absolute dose, local dose normalization, with 10% dose threshold (2/2) gamma criteria, was used for evaluation of these pass rates. This criteria was selected based on the suggestion by Masi et al.,^(17)^ where they found pass rates obtained with 2/2 tolerance are the most indicative of deliverable dose and plans with pass rates equal to or above 90% accurately represent the calculated dose from TPS.

**Figure 4:**
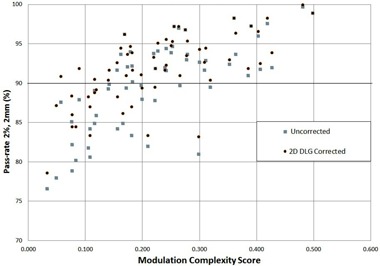
Gamma pass rates obtained for a 2%/2 mm gamma criteria as a function of Modulation Complexity Score (MCS). The data represent 61 VMAT fields comparing 2D DLG‐corrected delivered plan measured dose with TPS‐predicted dose and uncorrected delivered plan measured dose with TPS‐predicted dose.

Figure 4 shows the 2/2 gamma pass rates for all the clinical VMAT plans versus the degree of modulation based on the MCS value. This figure shows the pass rates for both 2D DLG‐corrected plans and the corresponding uncorrected standard plans. The uncorrected measurement points were obtained by calculating the 2/2 gamma pass rate between the delivered dose obtained from the uncorrected plan and the TPS predicted dose. The 2D DLG‐corrected points were obtained by calculating the pass rate between the delivered doses obtained from the same plans corrected for 2D variation of DLG and the TPS‐predicted dose. The 2D DLG‐corrected plans have higher pass rates than their corresponding uncorrected plans. The improvement in pass rates is most significant for highly modulated plans (low MCS score). Seventy‐one percent of the 2D‐corrected plans have pass rates higher than 90%, whereas only 56% of the uncorrected plans have pass rates higher than 90%. All the 2D DLG‐corrected fields with MCS score of higher than 0.22 had pass rates higher than 90%, whereas uncorrected fields with a MSC score value higher than 0.32 had pass rates higher than 90%. This indicates that the 2D DLG correction enables higher modulated plans to be more deliverable and accurately represent the calculated dose from TPS than the uncorrected plans.

**Figure 5:**
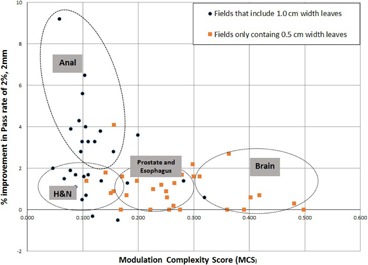
Improvement in pass rate after correcting the VMAT fields with 2D DLG variation map. Improvement in pass rate is compared against the Modulation Complexity Score. The solid circles represent fields employing both the 1.0 cm width MLC leaves and 0.5 cm width leaves, whereas the squares represent fields employing only the 0.5 cm width leaves.

Figure 5 shows the improvement in pass rate (2/2) after correcting the sample fields with 2D DLG map. It is evident from this figure that the higher the modulation (low MCS), the higher the improvement in pass rate after correction. This figure separates the fields employing both the 0.5 cm width MLC leaves and 1.0 cm width leaves (solid circles) and the fields employing only the central 0.5 cm width MLC leaves (solid squares). The anal fields employ almost all of the 1.0 cm width leaves with very high modulation (average MCS score of 0.124), hence, they

show the highest improvement in pass rates. The H&N fields are also very highly modulated with the average MCS score of 0.158. Unlike the anal fields, they only employ the innermost 1.0 cm width leaves to modulate the fluence. The improvement in pass rate for the H&N fields are not as high as the anal cases since most of the fluence is created by the 0.5 cm width leaves, where the DLG does not vary as much as the larger leaves; however, the correction is still significant. The prostate and the esophageal cases are moderately modulated with average MCS scores of 0.219 and 0.245, respectively. These fields are only modulated with 0.5 cm width MLC leaves, hence the improvement in pass rates is smaller. The brain fields have very low modulation, indicated by an average MCS of 0.350, and the improvement in pass rates is very small but still observable.

## IV. DISCUSSION

The fluences and the dose distributions produced by dynamic treatment fields shaped by the 1.0 cm leaf pairs need a 2D DLG correction to compensate for the lower DLG values of the 1.0 cm MLC leaf pairs. This is seen in the results obtained from the dynamic gap test plans. A large discrepancy between the uncorrected measured and TPS predicted doses, evident by high gamma indices, can be seen where the 1.0 cm leaves reside (Fig. 3). The 2D DLG corrected measurement shows very good agreement with the TPS‐calculated dose illustrating the need and importance of 2D DLG correction at these off‐axis distances.

For the 2 mm dynamic gap test field, Table 2 shows that the 2D correction improved the agreement between the measurement and TPS prediction by 23%, but the final pass rate is still very low (62.2%). This is because, as the MLC modulates, the random fluctuation in MLC positions can cause high dose differences for small gap fields, which cannot be accounted for by the 2D correction algorithm alone. Therefore, for dynamic fields with very small gap widths (~ 2 mm), the 2D DLG correction would improve the agreement between the predicted and the measured dose, but the pass rate may still be low.

As the gap size increases, these random fluctuations have decreased effect on delivered dose. In these cases, the 2D DLG correction improves the pass rate dramatically and can be seen in the gamma pass rates of the corrected test plans of 4 mm, 6 mm, and the clinical anal VMAT cases. The pass rates for the 2D‐corrected test plans are higher than 80%, whereas the uncorrected plans fall well below 80%. Most of the failures occur at points in the fluence created with 1.0 cm MLC leaf pairs (beyond the 10.0 cm superior and inferior from the CAX). The clinical anal cases, which utilize almost all of the 1.0 cm width leaves, showed the most improvement in pass rates, as evident from Fig. 5. The H&N cases had similar low MCS values as the anal cases, indicating high modulation, but did not show the same improvement in pass rates. This is because the H&N fields only employed the innermost 1.0 cm width MLC leaves with the 0.5 cm width leaves. Since most of the dose is delivered via the 0.5 cm width leaves, where the DLG does not vary much, the corrected and the uncorrected plans had similar pass rates.

For the 10.0 mm gap test fields, the pass rate improved by 7.7% when the 2D DLG correction was applied. This improvement is small compared the lower gap test fields, but still significant. As the effective MLC gap size increases beyond 1.0 cm, the 2D DLG could become less significant. This is also seen from the moderately modulated prostate and esophageal cases, as well as from the minimally modulated brain cases. 4, 5 show that the moderately modulated and the low modulated 2D DLG‐corrected clinical VMAT cases had only small improvement in pass rates (1% – 2%). For large MLC gaps, submillimeter differences in DLG values among leaf pairs may not greatly affect the calculated dose. In these situations, the approximation by the TPS in assuming a constant DLG for all the leaf pairs is valid, even for fluences created by the 1.0 cm width MLC leaves. For narrow dynamic MLC gap fields (<1.0 cm or MCS<0.32) utilizing the 1.0 cm width leaves, variation of DLG among leaf pairs become important in dose calculation and should be included in the TPS to model the MLC leaves more accurately.

With state‐of‐the‐art treatment techniques, such as SBRT‐VMAT and IMRT, high doses can be delivered to a target while minimizing doses to surrounding critical structures. As methods of delivery become more complex, the TPS also needs to evolve to predict these highly complex treatments accurately. One of the methods in achieving this goal is to incorporate a 2D variation of DLG. This will be useful for highly modulated treatment fields, especially those created by MLC of different thickness.

## V. CONCLUSIONS

The recent rapid increase in the quality of radiation therapy techniques enables the delivery to be very conformal to the target while sparing healthy tissue, but this increase in quality requires highly modulated treatment fields created by very complex MLC motions. Since the TPS algorithm assumes the MLC leaves have straight‐edged tips instead of curved tips, then uses the DLG measured for the middle MLC leaf pair for all the MLC leaf pairs, the predicted dose might not be same as that ultimately delivered to the patient. We have incorporated the 2D variation of DLG into several clinical VMAT cases with varying MLC motions characterized by the modulation complexity score. For fields with large MLC gaps, the 2D variation of DLG would only affect the delivered dose minimally. For fields with narrow dynamic MLC gaps, especially created by the outer 1.0 cm width leaves, the variation in DLG becomes important in dose calculation and should be included in the TPS to model MLC leaves accurately

## Supporting information

Supplementary Material
