# Supplementary figures and images for: Evaluation of fluence‐based dose delivery incorporating the spatial variation of dosimetric leaf gap (DLG)
Source: J Appl Clin Med Phys. 2016 Jan 8;17(1):12–21. doi: 10.1120/jacmp.v17i1.5883 (PMC5690211; doi:10.1120/jacmp.v17i1.5883)

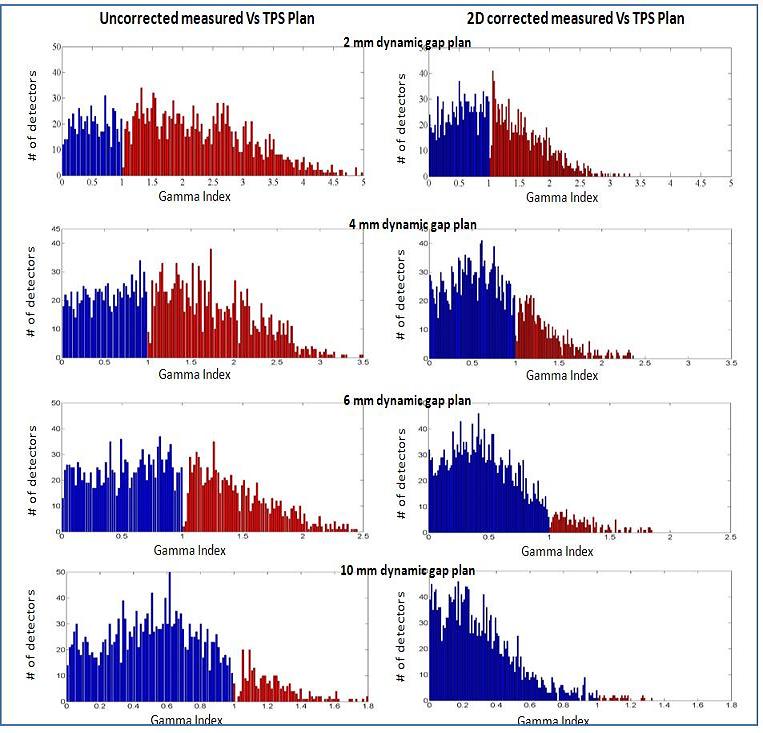

Supplement: Supplementary file 1 — Supplementary Material [file ACM2-17-012-s001.jpg]
